# The role of physical activity and fitness for children’s wellbeing and academic achievement

**DOI:** 10.1038/s41390-024-03467-y

**Published:** 2024-08-10

**Authors:** Julia Jaekel

**Affiliations:** 1 Psychology, University of Oulu, Faculty of Education and Psychology, Pentti Kaiteran katu 1, Oulu, 90014 Finland; 2 Department of Psychology, University of Copenhagen, Copenhagen, Denmark; 3 Department of Paediatrics I, Neonatology, Paediatric Intensive Care, Paediatric Neurology, University Hospital Essen, University of Duisburg-Essen, Essen, Germany; 4 Department of Psychology, University of Warwick, Coventry, UK

## Abstract

It is well known that physiological, psychological, and cognitive factors contribute to children’s wellbeing and school success, but studies assessing these domains simultaneously are surprisingly rare. Visier-Alfonso et al. expand on our existing knowledge base and report different pathways to academic achievement for girls and boys. Specifically, girls with higher cardiorespiratory fitness had better psychological wellbeing, and this was associated with higher academic achievement. Boys were more academically successful if they had higher cognitive flexibility. Boys with higher cardiorespiratory fitness also had better psychological wellbeing. According to this current evidence, cardiorespiratory fitness has both direct and indirect beneficial effects beyond physical health on psychological wellbeing and academic achievement. Health practitioners, education professionals, and parents should focus on increasing opportunities for daily physical activities that will benefit children’s cardiorespiratory fitness.

In today’s world, finding a good balance between screen time and physical activity is key to child health, wellbeing, and school performance. At least that is what most health practitioners, education professionals, and parents will likely agree on. However, despite the real-life importance of these domains, there is surprisingly little scientific evidence on how they are independently and simultaneously associated with each other. Study findings of how screen time affects child development and academic outcomes at school age have been mixed,^1,2^ especially when adjusted for families’ socio-cultural backgrounds and level of education. Visier-Alfonso et al. do not only expand on our existing knowledge base of how physiological, psychological, and cognitive factors contribute to children’s school success, they also provide new details on the strength (or their absence) of underlying associations. In their observational study of 519 school-aged children in Spain, the different domains were operationalised via well-established, reliable, multi-informant measures, e.g. cardiorespiratory fitness (CRF) was assessed with the 20-metre shuttle run test, recreational screen time use was reported by parents, psychological well-being was assessed from children themselves with the Kidscreen-27,^3^ and cognitive flexibility via the computerised Dimensional Change Card Sort Test.^4^ The selection and intentional combination of these measures allows a comparison of the current findings with other previous studies from different settings and world regions – a precondition for meaningful contributions to understanding developmental mechanisms.

Human development is shaped by complex multidirectional cascades over time.^5–7^ In research, it is important to design studies that allow us to include relevant variables and constructs in one model, in order to estimate and test hypothesised associations that mirror the true complexity of development. On the contrary, if relevant constructs and their associations are not included in statistical models, researchers risk overestimating certain direct associations by neglecting others. With regard to these methodological aspects, Visier-Alfonso et al.’s study is a step forward. They demonstrate how to apply fit indices provided by structural equation and path modelling to adapt hypothesised associations to a collected data sample. This data-based model fitting process is especially helpful when a sample is large enough to provide sufficient statistical power and assumed to be representative of a population.

Accordingly, Visier-Alfonso et al. report different pathways from CRF to academic achievement by biological sex, suggesting intriguing differences between girls and boys. Specifically, girls with higher CRF reported better psychological wellbeing, and this was associated with higher academic achievement. Boys, on the other hand, were more academically successful if they had higher cognitive flexibility. In addition, the authors report a total negative effect of screen time on academic achievement among boys, however it is small and only marginally significant. Boys with higher CRF also had better psychological wellbeing, but there were no associations of these variables with their academic achievement. These sex differences in associations between domains may be partly influenced by the current sample’s descriptive differences: on average, boys used screens more often and were more fit, but they had lower cognitive flexibility than girls. While these sex differences in mean values are in line with many other studies worldwide, the current results of different mechanisms still need replication in other samples and populations.

The oldest participants in the sample were 11 years at the time of data collection - on the cusp of adolescence. The fundamental hormonal and neurodevelopmental changes they will be undergoing throughout puberty will shape their physiological, psychological, and cognitive characteristics, and indirectly affect their future academic performance. Because of these changes, puberty represents a critical time of transition with a window of risk but also of opportunity: to set individuals on healthy trajectories of wellbeing and academic success. Visier-Alfonso et al.’s study provides pointers for some of the underlying mechanisms that may be changed through intervention during late childhood. The primary years of formal schooling trigger challenges for all children across multiple areas, including the expectation to pay attention and sit still for long periods of time, inhibit unwanted behaviours, and to self-regulate their own emotions, for example.^8,9^ In educational and developmental psychology research, children’s CRF, physical activity, and motor skills have traditionally been paid little attention to.^10,11^ However, these domains play an important role as part of the typical developmental cascades shaping preschool and early school age.^12,13^ Accordingly, in recent years, there has been a growing awareness of the critical role of visual-motor coordination and circumscribed motor coordination disorders, referred to as Developmental Coordination Disorder (DCD),^14–16^ as well as childhood obesity.^17^ Motor skills develop along a continuum in close association with other domains such as executive functions and social behaviour. For instance, coordination, balance, and handwriting involve complex skills ^15,18,19^ and are part of everyday activities at school. Difficulties with holding and moving a pencil, putting on shoes during lesson breaks, or clumsiness in group-based games can impact school performance and social participation. Not surprisingly, children’s motor abilities have been found to affect their self-esteem, well-being, acceptance by peers, and academic achievement.^18,20,21^ In the context of the current findings, CRF may be an indicator of children’s day-to-day levels of physical activity, which are not only paramount for motor skills and overall health but also play an important role in social interactions and inclusion in games among children. In Visier-Alfonso et al.’s models, the one and only stable and significant association across both sexes is the path from CRF to psychological wellbeing. This underscores that physical activity is universally foundational for participation and peer acceptance at school age, and thereby affects trajectories of long-term academic success and wellbeing.

After a close look at Visier-Alfonso et al.’s findings, the main takeaway is perhaps that health practitioners, education professionals, and parents should stress less about limiting screen time and instead focus on increasing opportunities for daily physical activities that will benefit children’s CRF. According to the current evidence, better CRF then has both direct and indirect beneficial effects beyond physical health on today’s children’s psychological wellbeing and academic achievement.
